# Carcinoma in a male accessory breast; Case report with literature review

**DOI:** 10.1016/j.ijscr.2025.110949

**Published:** 2025-01-24

**Authors:** Ahmed A. Almass, Mariyah E. Alzayer, Hussam J. Alsafwani, Alaa A. Salim, Mohammed Al Duhileb

**Affiliations:** a King Fahd Hospital of the University, Imam Abdulrahman Bin Faisal University, Khobar, Saudi Arabia; b General Surgery Department, Dammam Medical Complex, Dammam, Saudi Arabia; c Department of Pathology and Laboratory Medicine, King Fahad Specialist Hospital, Dammam, Saudi Arabia; d Breast and Endocrine Surgery Consultant, Johns Hopkins Aramco Healthcare, Dhahran, Saudi Arabia

**Keywords:** Accessory breast, Accessory breast cancer, Sebaceous cyst, Axilla, Axillary swelling

## Abstract

**Introduction and importance:**

Accessory breast is a rare condition where regression of the mammary ridge fails. This ectopic breast can function as the same pectoral breast and respond to hormonal effects. Furthermore, in rare cases, it can develop into malignancy. A malignant accessory breast is very rare, especially in male patients. Although it had first been reported in a male in 1957, there were only a few reported cases following that with no sufficient epidemiological data.

**Case presentation:**

A 60-year-old male with right axilla swelling is thought to be a benign lesion for excision. However, histopathology study of the specimen showed malignant cells, and a diagnosis of metastasis has been made and referred to our hospital. We did a full staging workup and eventually diagnosed him with primary accessory breast carcinoma, cT1N0M0.

**Clinical discussion:**

Diagnosis of male accessory breast cancer is challenging. Patients usually present with painless swelling. As it is very rare, the possibility of metastasis needs to be ruled out before the diagnosis is made. No current diagnostic and treatment guidelines for accessory breast cancer, and in current practice physicians follow the guidelines of regular breast cancer. Another difficulty with this disease is the estimation of its prognosis.

**Conclusion:**

Although accessory breast cancer is rare, it can be seen even in males. In patients with a lesion alongside the milk line, accessory breast cancer should be on the differentials list. Further studies regarding epidemiology, diagnostics, treatment plan, and prognosis of the disease need to be carried out.

## Highlights

• Accessory breast can function the same as the normal breast, and be affected by the same diseases.
• Accessory breast cancer is rare overall and exceptionally uncommon in the male population.
• Any swelling alongside the mammary line should raise the suspicion of accessory breast
• Accessory breast cancer is usually mistaken as a benign disease, especially in males.
• Although histopathology assessment is the confirmatory test of accessory breast malignancies, ultrasound has shown a good role in distinguishing them.

## 1 Introduction

Accessory breast, also called accessory mammary gland or ectopic breast, is a clinical condition where there is failure of regression of the embryonic mammary ridge, which is the milk line that starts from the axilla to the groin. This condition occurs in 1–3 % of males and 2–6 % of females (1). Accessory breast can include and act like a normal pectoral breast and be affected by hormonal changes. In India, a study showed that the most common symptom for axillary ectopic breast among women is secretions during pregnancy or lactation. Other reported symptoms included premenstrual pain and swelling (2). For it to develop into a malignancy is even rarer. To our knowledge, there are no comprehensive epidemiological studies regarding ectopic breast developing into malignancy, especially in male patients.

Noting that accessory breast tissue and sweat glands originate from the same stem cells, a study suggested that it is worth investigating if the malignancy is originating from breast tissue or apocrine tissue through histopathology for possible hormonal therapy (3). In males, it becomes extremely tricky to diagnose as it could present as vague non-specific symptoms such as: pain, erythema and swelling of the affected area (4). Therefore, the presentation of such cases is misleading to other possibly benign diagnoses and could lead to delayed or interrupted management.

We represent a 60-year-old male with adenocarcinoma in an accessory breast. This article has been written in line with the SCARE Criteria (5).

## 2 Case presentation

A 60-year-old Saudi male known to have diabetes, hypertension, dyslipidemia and renal impairment with no family history of malignancies referred to the breast oncology surgery clinic at a tertiary hospital as a case of metastatic axillary lymph node versus metastatic axillary nodule from an unknown source.

The patient was doing well until 1 year prior to the referral, when he was presented to a secondary hospital with a self-palpated axillary mass for 3 months. Clinical assessment showed a right axillary painless mobile subdermal 1*1 cm mass that had been diagnosed as a skin sebaceous cyst. It has been excised as a day case surgery under local anesthesia and sent for histopathology analysis. The histopathology results showed malignant cells, so they referred the patient to our clinic as a query breast metastasis to the axilla for further workups.

Upon physical examination at our clinic, we found gynecomastia grade I, left breast and axilla were unremarkable. The right breast and axilla were also unremarkable, except for the healed right axilla scar. The right upper limb was examined with no evidence of nevus.

**Fig. 1:**
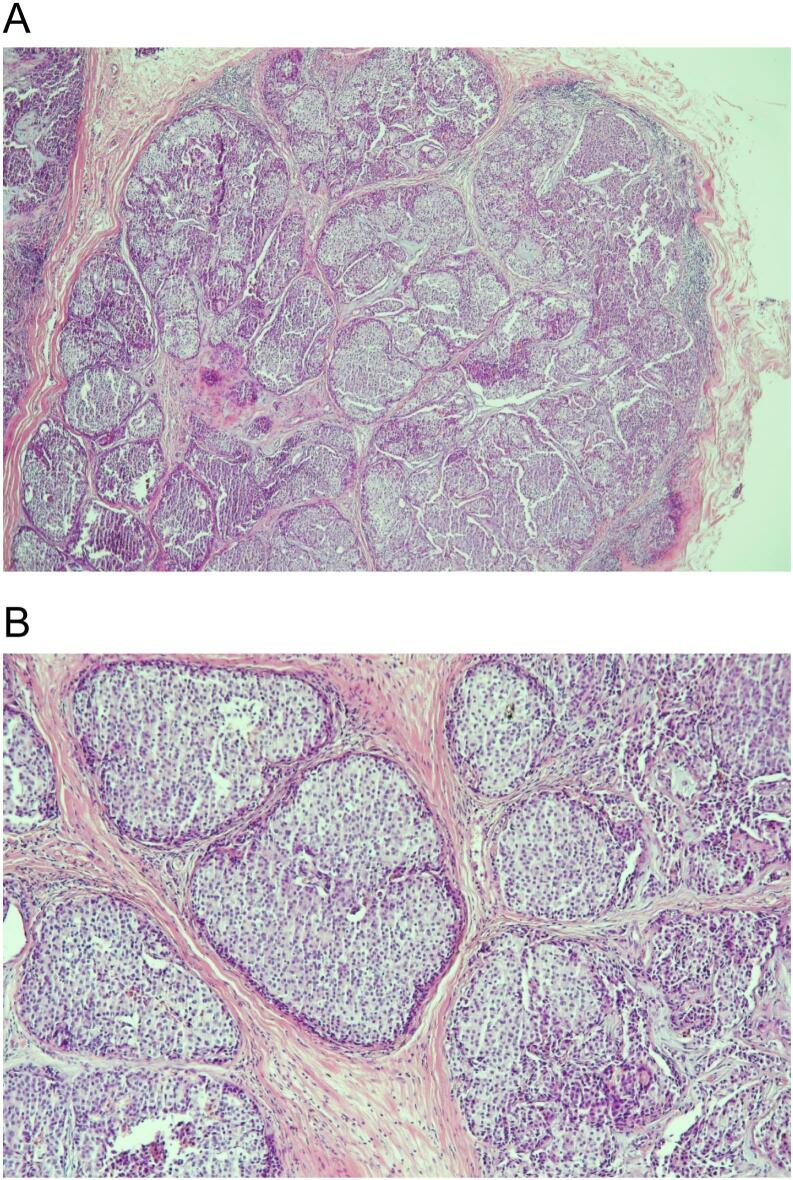
**A and 1B:** Adenocarcinoma favors breast origin with lympho-vascular invasion.

An attempt to find the origin of the mass. A breast mammogram and ultrasound were offered that showed normal bilateral breasts and axillae, BIRADS I, with no suspicious findings. A PET CT scan has also been carried out with no evidence of FDG avid malignancy. Review of the histopathology slides confirmed adenocarcinoma, favors primary breast origin with Grade 1 lympho-vascular invasion. It was positive for GATA-3, mammaglobin, GCDFP-15, ER, PR, and androgen receptors. The HER2NEU was negative (SCORE 1+) and the ki-67 was 10 % (see Fig. 1 and 1B).

As the pathology confirmed mammary origin with no lymphoid tissue in the specimen, and as the lesion is excised from the milk line, and no further lesions in the normal breast, we describe it as a primary accessory breast lesion. Our final diagnosis was right accessory breast invasive ductal carcinoma, luminal A, cT1N0M0 status post complete excision of the primary lesion in the axilla. As a male breast cancer, BRCA test was carried out and was negative. However, further, we did the oncology risk 89 genes and showed VOUS mutation in TP53.

Our multidisciplinary team decided to complete the assessment and staging with breast MRI and sentinel lymph node biopsy from the ipsilateral axilla, then to follow with the proper adjuvant therapy to ensure a full recovery. However, despite our efforts, the patient was in a denial state, refusing to continue his treatment then missed up from the clinic.

## 3 Discussion

A wide range of diseases can affect the axilla and present as axillary swellings. Lai, et al., in a pictorial essay classified the axillary swellings into nodal and non-nodal lesions that include surgery-related, infective or inflammatory and breast augmentation–related lesions. While the nodal lesions can be either benign or malignant, the non-nodal can originate from different tissues including, breast, skin, muscles and soft tissue, blood vessels, nerves, and bones (6).

Sebaceous cyst, also known as epidermoid cyst, is one of the common benign cutaneous cysts. It is a differential for axillary swelling. It is usually asymptomatic swelling unless it is inflamed. Although it is being diagnosed clinically or with the help of some images, its confirmation cannot be achieved without the help of histopathologic examination, and its management is accomplished by simple excision (7). Like in our case, histopathological assessment can help in ruling out or confirming other more serious diseases.

Another differential is accessory breast. In general, it is more noticeable among females, because it can act as a normal breast, which can be affected by hormonal changes and can be palpated. In a study in India among 30 women who had an accessory breast in their axilla, the most common complaint was swelling during pregnancy or during lactation (2). Male benign axillary breast is uncommonly reported in the literature. However, Bone et al. reported an interesting case of a male giant benign axillary breast (8).

Regarding breast cancer in males, it is less common compared with females as the risk is estimated to be around 1 in 800 compared to 1 in 8 for females, and the risk factors are similar to the ones in females (9). Men with BRCA 1/2 gene mutations are at increased risk of breast cancer, with a stronger association related to the BRCA 2 pathogenic variant accounting for 8 % compared to 1 % with the BRCA 1 mutation (10). ESMO Guidelines recommend annual screening for male patients carrying the BRCA 2 gene (11).

Additionally, for the BRCA 1/ 2 mutations, associations between breast cancer risk and truncating gene variants such as TP53, PTEN, NF1, Double carrier, CDH1 have been reported. Protein truncating variant TP53 is also associated with Li Fraumeni syndrome, with recommendation of annual MRI screening from the age of 20 for carriers of this gene (11). The lifetime breast cancer risk in patients with TP53 is higher than BRCA 1/ 2, which is estimated to be 80–90 % (12). Our patient apparently has no risk factors except for the TP53 mutation.

The incidence of malignancies in accessory breast is uncommon and ranges from 0.2 to 0.6 % (13). In males, it is extremely rare. Although it was first reported in 1957, a systematic review revealed that from 1987 to 2020 there were only 16 reported male cases in the literature and the majority were in China. Most of the involved cases did not receive medical management until after years of noticing the lesion, reaching 50 years in one case. Biopsy to rule out malignancies has been offered only in 4 of the 16 cases (1). From 2020, by reviewing the literature, more male cases can be identified ([14], [15], [16], [17], [18], [19]). This warrants further investigation of the epidemiology. In females, even though it is rare, there are quite a few case reports and literature behind it (4).

Regarding the presentation of malignant accessory mammary gland, it depends on the location of the gland. It could appear throughout the milk line from the axilla to the groin, where the axilla is the most common site, followed by the groin (1,20). The common presentation is slow growing swelling, irregular shape, non-tender and red on the unilateral axilla (20). Since the condition generally looks benign and does not interfere with daily activity, late presentation is common. Hence, the patient may present with pain, lymphadenopathy, erosion of the skin, or even purulent exudate (1).

Although there are no guidelines regarding the use of ultrasound, it has been used for assessment of the lesion in the literature, and the findings were hypoechoic mass with uneven echogenicity with irregular margins (21). Furtherly, the sebaceous cyst has typical findings in the ultrasound as well (7). Therefore, we suggest stakeholders consider the use of ultrasound in the assessment of axillary swellings. Unfortunately, in our case, ultrasound was not offered before the excision, and only found out to be malignant through histopathology.

The metastasis of breast cancer to the axillary lymph nodes is common. A study found it to be 25 % (22). Consequently, the confirmation of such lesions is made by ruling out the possibility of other origins and through histopathology assessment, as it would show normal breast tissue adjacent to the lesion besides the absence of lymphoid tissue (23). For staging, scientists recommend the use of some imaging modalities including MRI and PET CT, besides doing sentinel lymph node biopsy. In the systematic review mentioned above, lymph node invasion was common, as more than half of the cases showed invasion of at least one lymph node (1,20). Unfortunately, in our case, we only did a PET CT as the patient missed our clinic after offering the other modalities.

It is highly recommended that after establishing the diagnosis, the treatment should be the same as normal breast carcinomas, including the choice of adjuvant therapy. It is also recommended that the ipsilateral breast should not be targeted in treatment (24). Yet, the prognosis is difficult to predict due to the small available sample size and the limited follow-up data (25).

## 4 Conclusion

Accessory breast cancer is a rare condition, especially in males, often misdiagnosed as benign, leading to delayed treatment. Due to its rarity, no clear management guidelines exist. We recommend considering accessory breast cancer in lesions along the mammary ridge.

Further research is needed to establish specific diagnostic and treatment protocols, as well as for axillary swellings in general. For current management, physicians should follow the international updated guidelines.

## Author contribution

**Ahmed A. Almass:** study concept or design, data collection, data analysis or interpretation, writing the paper.

**Mariyah E. Alzayer:** study concept or design, data analysis or interpretation, writing the paper.

**Hussam J. Alsafwani:** study concept or design, data analysis or interpretation, writing the paper.

**Alaa A. Salim:** data collection, data analysis or interpretation.

**Mohammed Al Duhileb:** study concept or design, data collection, data analysis or interpretation.

## Informed consent

Written informed consent was obtained from the patient for publication and any accompanying images.

## Guarantor

Ahmed A. Almass.

## Sources of funding

This article is not funded by any funding agency, and not for profit purpose.

## Ethical committee approval

Ethical approval for this study was provided by the IRB Committee at our institution on 8 December 2024.

## Declaration of competing interest

The authors declare that they have no competing interests.
